# Significant Lifetime Enhancement in QLEDs by Reducing Interfacial Charge Accumulation via Fluorine Incorporation in the ZnO Electron Transport Layer

**DOI:** 10.1007/s40820-022-00970-x

**Published:** 2022-11-04

**Authors:** Dong Seob Chung, Tyler Davidson-Hall, Giovanni Cotella, Quan Lyu, Peter Chun, Hany Aziz

**Affiliations:** 1 Department of Electrical and Computer Engineering, Waterloo Institute for Nanotechnology, University of Waterloo, 200 University Avenue West, Waterloo, ON N2L 3G1 Canada; 2 Ipswich Research Centre, Huawei Technologies Research & Development (UK) Ltd., Phoenix House (B55), Adastral Park, Ipswich, IP5 3RE UK; 3 Ottawa IC Laboratory, Huawei Canada, 19 Allstate Parkway, Markham, ON L3R 5B4 Canada

**Keywords:** Colloidal quantum dots, Quantum dots light emitting device, Device stability, Zinc oxide nanoparticles, Fluorine

## Abstract

ZnO nanoparticles are widely used for the electron transport layers (ETLs) of quantum dots light emitting devices (QLEDs). In this work we show that incorporating fluorine (F) into the ZnO ETL results in significant enhancement in device electroluminescence stability, leading to LT50 at 100 cd m^−2^ of 2,370,000 h in red QLED, 47X longer than the control devices. X-ray photo-electron spectroscopy, time-of-flight secondary ion mass spectroscopy, photoluminescence and electrical measurements show that the F passivates oxygen vacancies and reduces electron traps in ZnO. Transient photoluminescence versus bias measurements and capacitance–voltage-luminance measurements reveal that the CF_4_ plasma-treated ETLs lead to increased electron concentration in the QD and the QD/hole transport layer interface, subsequently decreasing hole accumulation, and hence the higher stability. The findings provide new insights into the critical roles that optimizing charge distribution across the layers play in influencing stability and present a novel and simple approach for extending QLED lifetimes.

**Supplementary Information:**

The online version contains supplementary material available at 10.1007/s40820-022-00970-x.

## Highlights

- Quantum dots light emitting devices (QLEDs) with extremely long half-lifetimes at 100 cd m^−2^, 2,370,000 h were successfully fabricated using CF_4_ plasma-treated ZnO nanoparticle electron transport layers.
- A new experimental approach that probes changes in exciton lifetime under current flow was used to investigate the changes in carrier concentration in QLEDs.
- Evidence of the dependence of QLED stability on electron and hole concentrations at the QD/HTL interface was revealed.

**Supplementary Information:**

The online version contains supplementary material available at 10.1007/s40820-022-00970-x.

## Graphical abstract

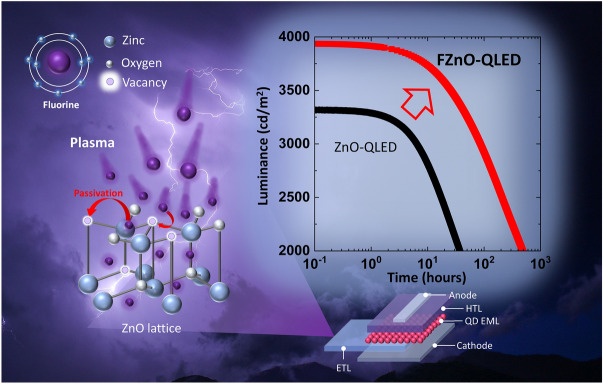

## Introduction

Ever since its benefits for the efficiency of quantum-dot light emitting devices (QLEDs) have been demonstrated [1], zinc oxide (ZnO) has become the material of choice for their electron transport layer (ETLs) almost universally [2–6]. Because of their amenability to solution-processing and good electrical properties, ZnO nanoparticles (NPs), nano-size particles dispersed in a solvent solution, are commonly utilized in making these ETLs in QLEDs as well as in various other electronic [7, 8] and optoelectronic devices [4, 9, 10]. External quantum efficiencies (EQE) exceeding 20% have been demonstrated in QLEDs with ETLs made of ZnO NPs [11, 12].

In QLEDs, charge concentrations and their build-up in the layers bulk and at interlayer interfaces directly influence excitonic phenomena and the efficiency of the radiative processes [11–14]. Therefore, optimizing charge injection and transport in the QD light emission layer (EML) and the charge transport layers is crucial for device efficiency and stability. From an energy bands standpoint, the well-matched conduction band minima (CBM) of ZnO and QD facilitate efficient electron injection into the QD EML to form excitons, assisted by Coulomb interactions with holes supplied by hole transport layers (HTL). Stoichiometric and structural defects in ZnO, however, lead to sub-bandgap states that make it difficult to accurately estimate and optimize charge injection at the interface on the basis of CBM offsets alone [15–17]. Additionally, asymmetric charge injection and transport in the ETL versus the HTL makes reaching optimal electroluminescence performance in these devices difficult. The lack of a complete and accurate understanding of these interfacial phenomena often leads to contradictory findings when attempting to resolve performance issues in QLEDs [18–22]. Approaches for modifying and optimizing the ZnO/QD interface are therefore often attempted. The use of polymers, either in the form of an interfacial layer in between ZnO and QD [22–27] or blended with ZnO across the entire ETL [20, 28] has been found to sometimes help optimize the interface. Chemical treatments and additives for modifying the structural and electrical properties of ZnO are also often used [29–32]. In this regard, doping ZnO with Mg has been found to be particularly effective for optimizing charge injection and transport, making it possible to realize high device efficiencies [33, 34]. Most highly stable QLEDs, however, utilize undoped ZnO [35].

In this work we investigate the influence of incorporating fluorine (F) into the ZnO NP ETLs on QLED performance. Doping by F has been found to modify ZnO properties in the past [36, 37]. Furthermore, the ionic radius of F is comparable to that of oxygen, making it possible to neutralize oxygen vacancies without causing significant lattice distortions [38–40]. While the use of F for passivating ZnO oxygen vacancies has been pursued in thin film transistors [40, 41] and photovoltaic devices [38, 39, 42, 43], it has not yet been investigated for QLEDs. Here we find that incorporating F into the ZnO ETLs of QLEDs significantly improves their electroluminescence stability. Via the use of carbon tetrafluoride (CF_4_) plasma-treated ZnO (FZnO) NP ETLs, we successfully fabricated highly stable inverted red QLEDs exhibiting 2,370,000 h electroluminescence half-life at an initial luminance of 100 cd m^−2^, 47 times longer than their counterparts with the ZnO NP ETL (without the F). The devices also exhibited 15% higher EQE. Investigations show that the incorporation of F into the ZnO ETL affects carrier distribution throughout the QLED structure, in turn affecting the electron–hole (e–h) recombination zone and rate in the QD EML and at the QD/HTL interface. The results underscore the influential role that modulating electron and hole concentrations near the QD/HTL interface plays in QLED stability.

## Experimental Section

### Device Fabrication

Indium tin oxide (ITO) patterned glass substrates (Kintec) are carefully cleaned and sonicated by Micro 90 cleaning solution (Cole-Parmer) and de-ionized water followed by sequential rinsing by acetone and isopropanol. The cleaned substrates are treated by oxygen plasma to improve surface wettability. 30 mg mL^−1^ ZnO NP dispersed solution (SkySpring Nanomaterials, Inc.) is spin-coated at 3,000 rpm on the cleaned ITO substrates followed by 400 °C baking on a hotplate for 30 min. CF_4_ plasma modification is performed in ICP-RIE chamber (Phantom RIE, Trion technology) filled with 20 sccm of CF_4_ gas and 20 W with 13.56 MHz operation frequency for 60 s. 13 mg mL^−1^ CdZnSe/ZnSe/CdZnS/ZnS QDs (Mesolight Inc.), of 75% photoluminescence quantum yield (PLQY), dispersed in octane (Sigma-Aldrich) are deposited on ETL by spin-coating with 3,000 rpm followed by post-baking on a 50 °C hotplate for 30 min. 50 nm of 4,4’-bis(*N*-carbazolyl)-1,1’-biphenyl (CBP, Angstrom Engineering), 5 nm of molybdenum trioxide (MoO_3_, Angstrom Engineering) and 100 nm of aluminum (Al, Angstrom Engineering) are deposited by a thermal evaporator (Angstrom Engineering) under 2 $\times$ 10^–7^ Torr condition. Overall device structures are: ITO/ETL/QD/CBP (HTL)/MoO_3_ (hole injection layer)/Al. All deposition processes are performed in nitrogen-filled glove box and vacuum chamber.

### FIrpic Marking Layer Device Fabrication

A 5 nm of CBP (95%):FIrpic (5%) marking layer is deposited on ITO/ETLs/QD by evaporating CBP and FIrpic from different sources with the evaporation rates individually controlled to obtain 0.95 and 0.05 Å s^−1^, respectively. A 45 nm of CBP is then deposited to obtain a HTL of a total thickness of 50 nm.

### Device Characterization

X-ray photoelectron spectroscopy (XPS) measurement are performed by VGS ESCALab 250 system with Al Kalpha X-ray source. TOF–SIMS depth profile is measured by IonTOF-SIMS-5. Current density–voltage-luminance (*J-V-L*) characteristics are measured by a Minolta CS-100 chromameter and an Agilent 4155C semiconductor parameter analyzer connected with a silicon photodiode. EL and PL spectra are measured by an Ocean Optics QE65000 spectrometer with an excitation source consisting of a Newport 67,005 200 W HgXe arc lamp and monochromator. Device luminance *vs*. time driven with constant currents are recorded using a M6000PLUS OLED lifetime test system. Surface topography measurements are performed using a Veeco Nanoscope tapping-mode atomic force microscope (AFM). Time-resolved photoluminescence (TRPL) is measured using an Edinburgh Instruments FL920 spectrometer connected to a time-correlated single photon counting unit (TCSPC) using a pulsed LED laser as an excitation source. *C-V* characteristics are measured using a Keithley 4200 semiconductor analyzer with a capacitance–voltage unit (4215-CVU). The capacitance is measured using an AC voltage with 20 mV room-mean-square and 5 kHz frequency. The small AC voltage signal produces current that is integrated to calculate *C* using the relationship (Eq. (1)):

1 $$C \equiv \frac{\Delta Q}{{\Delta V}}$$

## Results and Discussion

### Chemical Composition Changes of Fluorine Plasma-treated ZnO NP

**Fig. 1:**
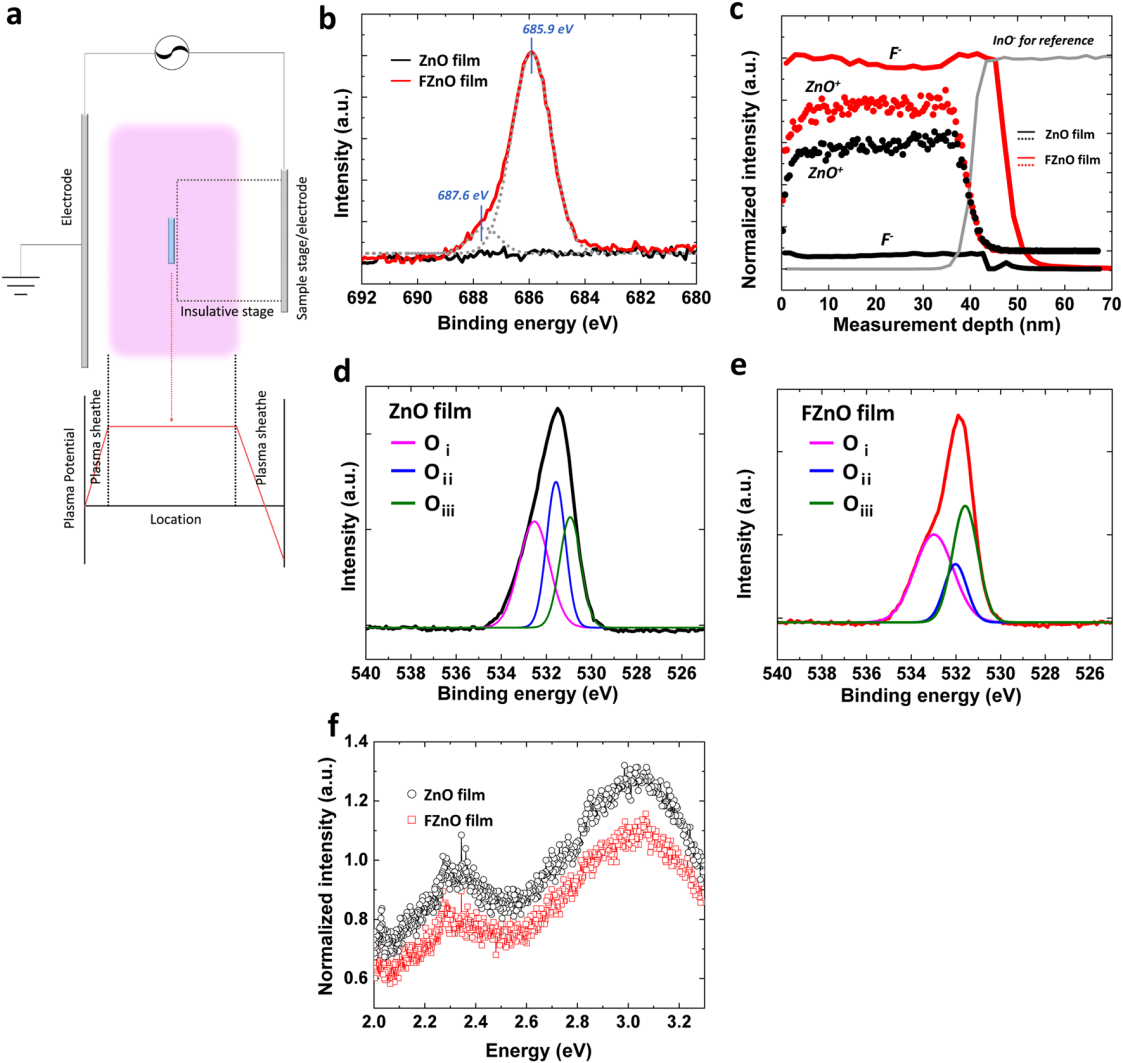
**a** Schematic diagram of the plasma treatment setup. XPS spectra of F 1*s* core level electrons in **b** ZnO and FZnO films and **c** F^−^ and ZnO^+^. All intensity values of TOF–SIMS are normalized by intensity of InO^−^ signal. O 1*s* core level electrons in **d** ZnO film and **e** FZnO film. **f** Steady state PL spectra under 330 nm UV excitation collected from the ZnO and FZnO films

ZnO NP films are subjected to a F plasma using a parallel plate reactor with a CF_4_ source gas. In a conventional parallel plate reactor, the sample to be treated is typically placed on a stage on one of the two electrodes. The high potential gradients near the electrodes, however, lead to the acceleration of charged plasma species and their collision with the sample surface at high kinetic energies resulting in bond cleavage and damage to the sample [44]. Therefore, to avoid this effect, we instead place the samples in the middle of the plasma chamber where the potential gradients are smaller and ion bombardment effects are minimal, as schematically depicted in Fig. 1a. Chemical modification of the ZnO NPs by the CF_4_ plasma treatment is verified by XPS. Figure 1b depicts the XPS spectra of F 1*s* core level electrons for FZnO and non-exposed ZnO films. To remove any surface contamination, Ar sputtering for 30 s is conducted on the film surface before the XPS measurement. A distinguishable F 1*s* spectrum emerges in the plasma-treated film (i.e., FZnO) that is not observed in the untreated ZnO film. The emergent F 1*s* spectral peak can be deconvoluted into two bands with maxima at 685.9 and 687.6 eV [39]. The 685.9 eV band can be ascribed to Zn-F bonding whereas the 687.6 eV band may be attributed to elemental fluorine or to F–C species [45] (with C present in the surface ligands of the ZnO NPs, for example). Fluorine, zinc and oxygen ion depth profiles in ZnO and FZnO films are further investigated through time-of-flight secondary ion mass spectroscopy (TOF–SIMS) in Fig. 1c. The peak intensities of the detectable species in Fig. 1c are normalized to the intensity of the indium oxide ions. Clearly, negative fluorine ion species are detected throughout the entire FZnO film thickness but not in the ZnO film. This observation confirms that the plasma treatment leads to F incorporation across the entire bulk and is not limited to the film surface. Figure 1d, e shows XPS band spectra of O 1*s* core level electrons in ZnO and FZnO films, respectively, again collected after a 30 s Ar sputtering to remove any surface contaminants (XPS spectra from the same samples before the sputtering are presented in Fig. S1b, c). The highest (*O*_*i*_) and lowest (*O*_*iii*_) energy bands correspond to hydroxyl groups and ionized oxygen ions, respectively, whereas the middle one (*O*_*ii*_) can be attributed to electronic states associated with oxygen vacancies in the lattice [38]. Quite notably, the relative intensity of the O_*ii*_ band decreases in case of FZnO film. This suggests that the fluorine atoms passivate the oxygen vacancies in the ZnO lattice, possibly by bonding with unbonded Zn^2+^ states. Steady state photoluminescence (PL) spectra under 330 nm UV excitation for the ZnO and FZnO films were also collected and are shown in Fig. 1f. There are two distinguishable bands at 2.3 and 3.1 eV, which can be ascribed to emission from Shockley–Read–Hall (SRH) recombination at sub-bandgap states [46, 47], and originating from oxygen vacancies and other defects such as zinc interstitials which lead to different states in ZnO films, respectively [48–50]. The PL intensity of both bands becomes lower in FZnO, an effect similar to that observed in Mg-doped ZnO [51], suggesting that the presence of the fluorine passivates the sub-bandgap states and reduces radiative electron–hole recombination.

### QLED Electroluminescent Characteristics

**Fig. 2:**
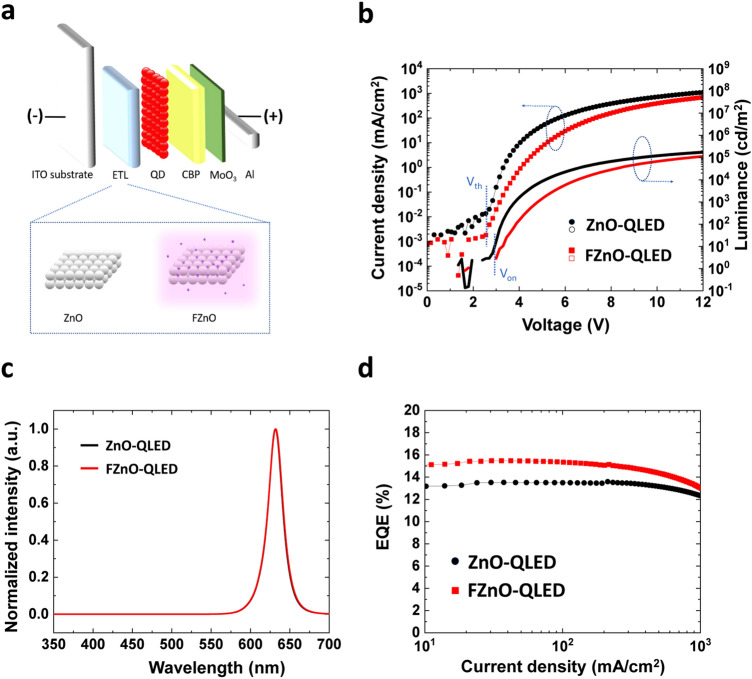
**a** Schematic diagram depicting the QLED structure. **b** *J-V-L* characteristics, **c** EL spectra, and **d** EQE *vs.* current density of QLEDs with ZnO *vs.* FZnO ETLs

Seeing that the F incorporation may be passivating the sub-bandgap states in the FZnO NP film, one can expect the use of FZnO as ETL in QLEDs may improve device performance. FZnO NP films are therefore incorporated as ETLs in inverted red QLEDs to investigate their effect on the electroluminescent (EL) characteristics. The structure of the QLEDs is schematically shown in Fig. 2a, and consists of, in order: ITO/ETL (40 nm)/QD (30 nm)/ 4,4’-bis(*N*-carbazolyl)-1,1’-biphenyl (CBP) (50 nm)/MoO_3_ (5 nm)/Al. Two groups of QLEDs of this general structure but with different ETLs are fabricated: ZnO (control device, ZnO-QLED) and FZnO (CF_4_ plasma-treated, FZnO-QLED). The EL characteristics of the QLEDs are shown in Fig. 2b–d. The shown characteristics represent typical values from among a set of 9 devices from each group fabricated on different days. The *J-V-L* characteristics in Fig. 2b show that the FZnO-QLEDs have similar threshold voltage (*V*_th_) and turn-on voltage (*V*_on_) to the ZnO-QLEDs. The current density at any given voltage is, however, lower indicating that electron transport is affected by the treatment. Another notable point is the reduction in current density below *V*_th_ in case of the FZnO-QLEDs. The sub-threshold current reduction can be explained in terms of the current flow mechanism in the low electric field regime, which for a junction-based diode structure, will be dominated by carrier recombination and described by Eq. (2) [52, 53],

2 $$J_{{{\text{rec}}}} = \frac{{q \cdot n_{i} \cdot t_{{{\text{scr}}}} }}{{\tau_{{\text{r}}} }}$$

where *J*_rec_ represents the recombination current density, and *q*, *n*_*i*_, *t*_scr_, and *τ*_*r*_ represent the elementary charge, intrinsic carrier density in the material, the width of space charge region in the junction, and recombination lifetime, respectively. Since the recombination process involves both band-to-band recombination ($\tau_{{\text{band to band}}}$) and SRH recombination ($\tau_{{{\text{SRH}}}}$), *τ*_*r*_ will be given by Eq. (3):

3 $$\frac{1}{{\tau_{r} }} = \frac{1}{{\tau_{{\text{band to band}}} }} + \frac{1}{{\tau_{{{\text{SRH}}}} }}$$

where $\tau_{{{\text{SRH}}}}$ is given by Eq. (4):

4 $$\tau_{{{\text{SRH}}}} = \frac{1}{{\alpha_{{{\text{SRH}}}} \cdot N_{{\text{t}}} }}$$

with $\alpha_{{{\text{SRH}}}}$ and $N_{{\text{t}}}$ representing the SRH recombination coefficient and trap density of states, respectively. Since, based on the PL results, SRH recombination must be less in case of the FZnO-QLED, the lower current density at voltages below *V*_th_ can be ascribed to the lower trap density, in line with Würfel et al. [52] and Anderson et al. [53] The small difference between *V*_th_ and *V*_on_ indicates that carriers are efficiently injected for exciton formation and radiative recombination in the QD EML. EL spectra of the ZnO- and FZnO-QLEDs are shown in Fig. 2c. Both devices exhibit strong QD emission at 630 nm without any discernable parasitic emission from the other layers. The EQE *vs.* current density characteristics of the QLEDs are shown in Fig. 2d. The FZnO-QLED shows a maximum EQE of 15.3%, a 15% enhancement relative to the ZnO-QLED control device. Given that the PLQY of the QDs used in this work is 75%, and based on a 20% out-coupling efficiency, the EQE of FZnO-QLEDs approaches the theoretical limit for a device with these QDs pointing to optimal charge balance and minimal exciton quenching conditions. With the decrease in sub-bandgap states in ZnO by the fluorine treatment, the efficiency improvement may be attributed at least in part to the passivation effect on ZnO trap states which can act as quenching centers.

### QLED Luminance Lifetime Enhancement

**Fig. 3:**
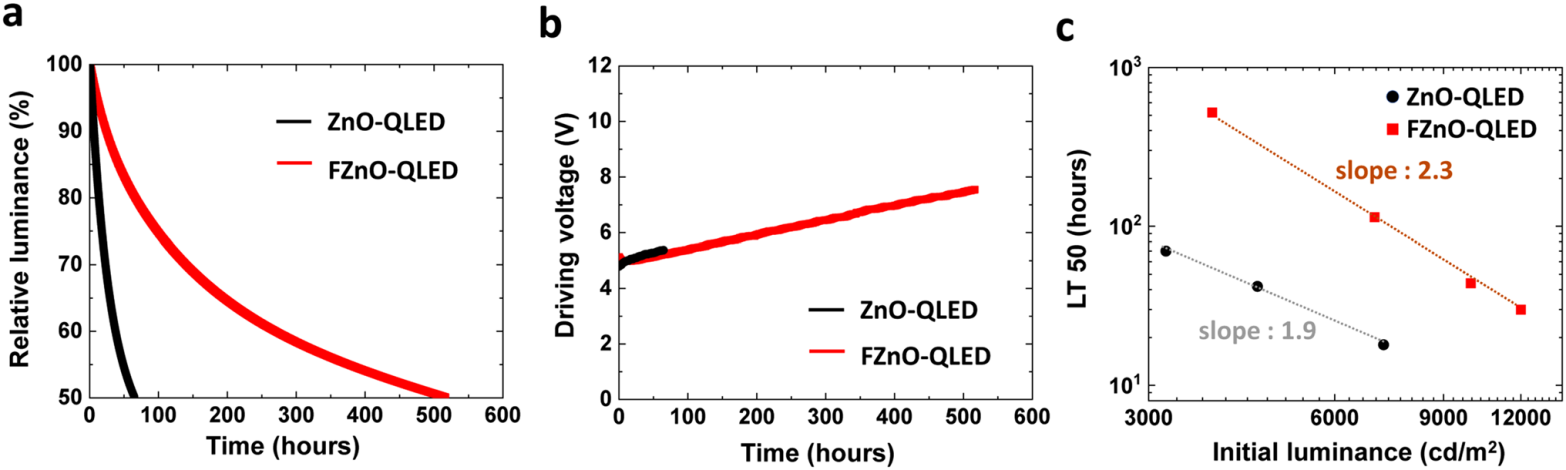
**a** Luminance decay *vs.* time and **b** driving voltage *vs.* time of ZnO- and FZnO-QLEDs with ZnO and FZnO ETLs operated at a constant current density of 20 mA cm^−2^. **c** Measured LT50 values for different devices with operated at different L_0_ values. The slopes correspond to the accelerator coefficients

In addition to the improvement in EQE, the FZnO-QLED has been found to exhibit a significantly higher EL stability over the ZnO-QLED control. Luminance and driving voltage evolution versus time under constant current at 20 mA cm^−2^ are presented in Fig. 3a, b, respectively. The initial luminance (*L*_0_) is 3,320 and 3,910 cd m^−2^ for the ZnO- and FZnO-QLEDs, respectively. The LT50s (the time for luminance to reach 50% of L_0_) of ZnO-QLED and FZnO-QLED are found to be 65 and 516 h, respectively. In addition, both QLEDs show negligible difference in driving voltage evolution in Fig. 3b. Different from some of the other reports for highly stable QLEDs via ETL modification where an exponential-like increase in driving voltage is sometimes observed [20], the FZnO-QLED shows a more linear trend which is more advantageous for long-term device operation. For a more accurate comparison between the lifetime of the devices, the LT50 equivalent values for *L*_0_ of 100 cd m^−2^ is calculated using the lifetime scaling equation (Eq. (5)) [54]:

5 $${ }L_{0}^{n} LT50 = constant$$

where *n* is an accelerator coefficient. The value of *n* is calculated to be 1.9 and 2.3 for QLED with ZnO and FZnO, respectively, based on stability tests on different devices at various *L*_0_ values as depicted in Fig. 3c. The different lifetime scaling coefficient values suggest that there may be an underlying difference in the degradation mechanisms of ZnO- and FZnO-QLEDs. The projected LT50 for *L*_0_ = 100 cd m^−2^ of the FZnO-QLED corresponds to 2,370,000 h, a QLED lifetime improvement of 47 times over the 50,000 h for the ZnO-QLED. To the best of our knowledge, this represents the longest LT50 for QLEDs utilizing conventional core/shell QDs obtained by ETL modification, suggesting that further stability enhancements may be possible if customized core/shell QDs or HTL materials are used with this ETL [35, 55]. Table S1 summarizes data from previous reports of QLEDs with improved stability achieved via ETL modifications.

### Defect passivation Induced Electron Concentration Increases in QD

**Fig. 4:**
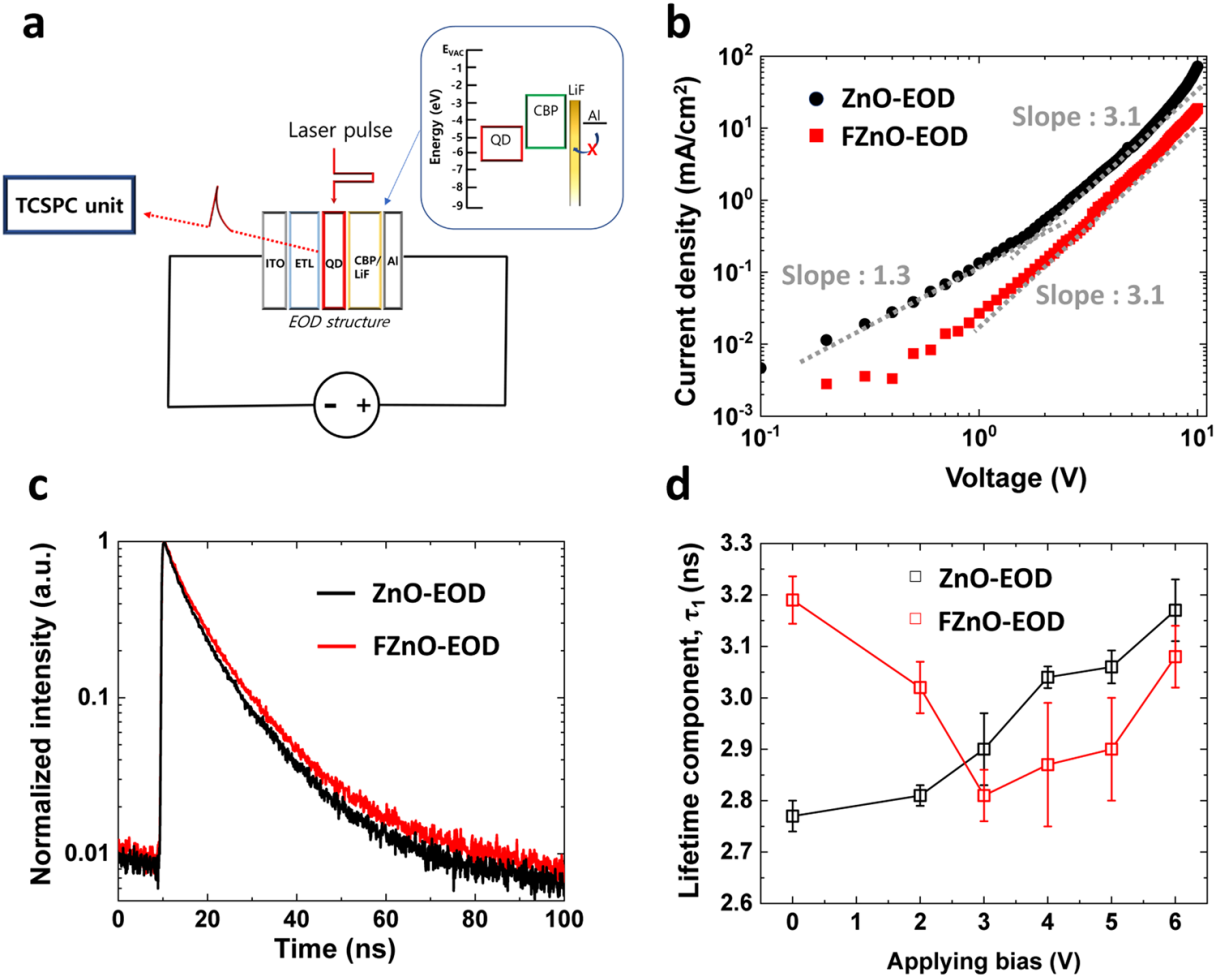
**a** Schematic diagram of the EOD structure and the TRPL measurements scheme. The inset shows the energy levels of QD/CBP/LiF/Al. **b** *J-V* characteristics of EODs with ZnO and FZnO. **c** TRPL signal from the QD layer in the EODs with ZnO and FZnO without bias. **d** The τ_1_values extracted from the TRPL signal under the different bias levels. The error bars indicate the standard deviation in the data collected from 6 samples in each group

While the F incorporation in ZnO via the CF_4_ plasma treatment clearly leads to a significant enhancement in device stability, it is unlikely that the enhancement is due to an increase in the chemical stability of the ETL. This is because ZnO is already thermodynamically stable [9, 18, 56]. In addition, since the surface of the ZnO and FZnO films are both hydrophilic (both exhibiting a water contact angle below 90 degree) and have somewhat similar surface topography (as characterized in Fig. S2), it is similarly unlikely that the stability enhancement is due to differences in the surface properties of the ETLs. We therefore suspect the effect might be associated with changes in exciton relaxation dynamics in the QD EMLs due to the passivation of ZnO surface defects or in charge distribution across the QD EMLs. It is known that a change in charge distribution in the QD EMLs could affect device stability [20]. The electron transport characteristics of the FZnO *vs.* ZnO ETLs are therefore studied and compared in electron-only devices (EODs). Additionally, time-resolved photoluminescence measurements (TRPL) are used to study and compare the influence of electron currents on QD exciton lifetime. Figure 4a depicts a schematic diagram of the experimental setup. In order to avoid the injection of holes from the Al electrode when under bias, and, at the same time, minimize—as much as possible—perturbations that may arise from differences in device structure between the EODs and the actual QLEDs, the same layer stack composition is used in the EODs as was used in the QLEDs except that the MoO_3_ layer was replaced by a 3 nm thick LiF layer in order to block hole injection into CBP [20]. The inset in Fig. 4a illustrates the energy levels of the QD/CBP/LiF/Al portion of the EOD, showing the large hole injection barrier behind the hole-blocking nature of the contact. We also wish to note that the devices do not emit any detectable EL over the voltage range of the measurements proving that hole injection is indeed insignificant. Figure 4b depicts and compares the *J-V* characteristics of ZnO-EOD and FZnO-EOD. The characteristics suggest an ohmic contact at the ETLs/QD EML interface which may explain why the threshold voltage of the QLEDs (in Fig. 2b) was not significantly changed. As can also be seen, the current at any given voltage is lower in case of the FZnO-EOD suggesting that the presence of fluorine reduces electron conduction across the ETL, which can be ascribed to the passivation of oxygen vacancies [16] and is consistent with the changes in the *J-V* characteristics of the QLEDs observed in Fig. 2b. The characteristics also have somewhat different shapes pointing to different charge transport behaviors. In case of the ZnO-EOD, the characteristics show a well-defined inflection point at around 2 V. The FZnO-EOD characteristics, in contrast, show what could be a much less defined inflection point at below 0.5 V, although, given the lower current levels in this case (below 1 µA cm^−2^) instrument limitations can make the measurements accuracy less certain. The clear change in slope at the inflection point – at least in case of the ZnO-EOD – points to a change in the dominant carrier transport mechanism between the low and high voltages [57–59]. At voltages below 2 V, the ZnO-EOD characteristics exhibit a slope of 1.3 suggesting trap-limited space charge limited current. In contrast, at voltages above 2 V, the characteristics of both devices exhibit a slope of 3.1 suggesting conduction becomes dominated by trap-filled carrier transport. In general, the transition voltage, *V*_*TF*_, at which the transition from trap-limited to trap-filled carrier transport occurs will depend on Eq. (6):

6 $$V_{{{\text{TF}}}} = \frac{{q \cdot N_{t} \cdot t}}{{2\varepsilon_{r} }}$$

where *t* and *ε*_*r*_ represent the layer thickness and dielectric constant, respectively, and *N*_*t*_ represents the trap density, which in our ETLs scales with the density of oxygen vacancies. The higher *V*_*TF*_ in the ZnO-EOD relative to the FZnO-EOD implies that a larger number of trap states (i.e., oxygen vacancies) exists in ZnO than in FZnO, and is consistent with the defect passivation effect of the fluorine observed in Fig. 1d-f.

With knowledge that the oxygen vacancies in ZnO are filled via fluorine incorporation, the effect of electron injection on QD lifetime was subsequently evaluated and compared in the EODs. In order to accomplish this, TRPL measurements on the QD layers in the EODs were conducted at the QD peak emission wavelength of 630 nm via excitation with a 380.2 nm laser pulse while a bias was simultaneously applied to the device as illustrated in Fig. 4a. In general, the decay in PL intensity over time PL(t) subsequent to the excitation can be deconvoluted into multiple exponential decay components each having a lifetime ${\tau }_{n}$ that is characteristic of the underlying exciton deactivation process according to the curve fitting equation (Eq. (7)):

7 $$PL\left( t \right) = \mathop \sum \limits_{n = 1}^{3} A_{n} {\text{exp}}\left( {t/\tau_{n} } \right)$$

where $A_{n}$ is a weighting coefficient. Table S2 gives the extracted values for the *τ* and *A* parameters assuming a tri-exponential decay rate. In QDs, the fastest exciton deactivation process is typically dominated by non-radiative recombination at the layer interfaces [22, 60] as well as by negative trion decay [61–63]. The τ_1_ values are therefore the most relevant for this analysis. In the absence of an external bias, the TRPL measurements show a lower exciton decay rate (in Fig. 4c) and a larger τ_1_ value (in Fig. 4d) in case of the FZnO, evidence of a reduction in the non-radiative deactivation of excitons by defects at the ETL/QD in the treated samples, consistent with the above results. Given its influence by negative trion decay, one can expect τ_1_ to also be strongly affected by electron injection. We therefore also do TRPL measurements on the EODs under forward bias (i.e., with the ITO held at a more negative potential relative to the Al contact) to investigate the effect of current flow on exciton decay rate. The approach taken here is different from that sometimes followed in other reports where TRPL measurements are carried out under reverse bias or with blocking contacts for the purpose of investigating the effect of electric fields—without any current flow—on exciton lifetime [64, 65]. As can be seen in Figs. 4d and S3, applying the forward bias leads to changes in QD exciton lifetimes and τ_1_ values. Notably these changes are quite different in the two devices, with the increase in voltage leading to a progressive increase in τ_1_ in case of the ZnO-EOD but an opposite trend initially in case of the FZnO-EOD before the trend reverses and τ_1_ also starts to increase with bias although to a lesser extent than in the control device. An increase in τ_1_ with bias is opposite to what one would expect from the familiar phenomenon of electric field-induced exciton dissociation and shows that the observed changes in τ_1_ are indeed current-induced rather than field-induced. It should be also noted that applying a reverse bias was found to have a negligible effect on τ_1_ (as can be shown in Fig. S4), further proving that the changes observed under the forward bias are driven by the current flow rather than by the fields. Considering that carrier transport is trap-limited in these ETLs, filling the traps in ZnO with electrons may contribute to the increase in τ_1_ in the ZnO-EOD. In other words, the continuous supply of electrons from the cathode neutralizes the oxygen vacancies in ZnO leading to a reduction in exciton dissociation at the ZnO/QD interface. In contrast, since a large fraction of the trap states is already passivated by the fluorine, the flow of current leads to a more modest increase in τ_1_ in the FZnO-EOD and only at higher voltages (> 3 V) when current levels become significant. The initial decrease, rather than increase, in τ_1_ observed at the low voltages in this case might stem from the presence of the faster decaying channel from negative trions whose concentrations in the QD layer of the FZnO-EOD can be expected to be higher due to the higher electron concentrations from the lower onset of reaching trap-filled transport in case of FZnO. As these trions become easily dissociated at high electric fields, their influence on τ_1_ subsides at the higher voltages (i.e., at > 3 V or 300 kV cm^−1^) [66]. The presence of a large number of injected electrons at the higher voltages increases their electric field screening effects which may reduce the field-induced trion dissociation, hence the late onset of this effect. Regardless of the specific mechanism, the different bias-dependence of exciton lifetime in the two EODs indicates that replacing ZnO with FZnO as the ETL changes electron distribution in the device stack possibly leading to higher electron concentrations in the QD layer near the QD/HTL interface. The higher electron concentration within the QD EML when using FZnO may be behind the significantly higher stability and efficiency of these QLEDs. The differences in exciton quenching and carrier distribution between the FZnO-EOD and the ZnO-EOD are schematically illustrated in Fig. S5.

### Electron and Hole Recombination at the QD/HTL Interface

**Fig. 5:**
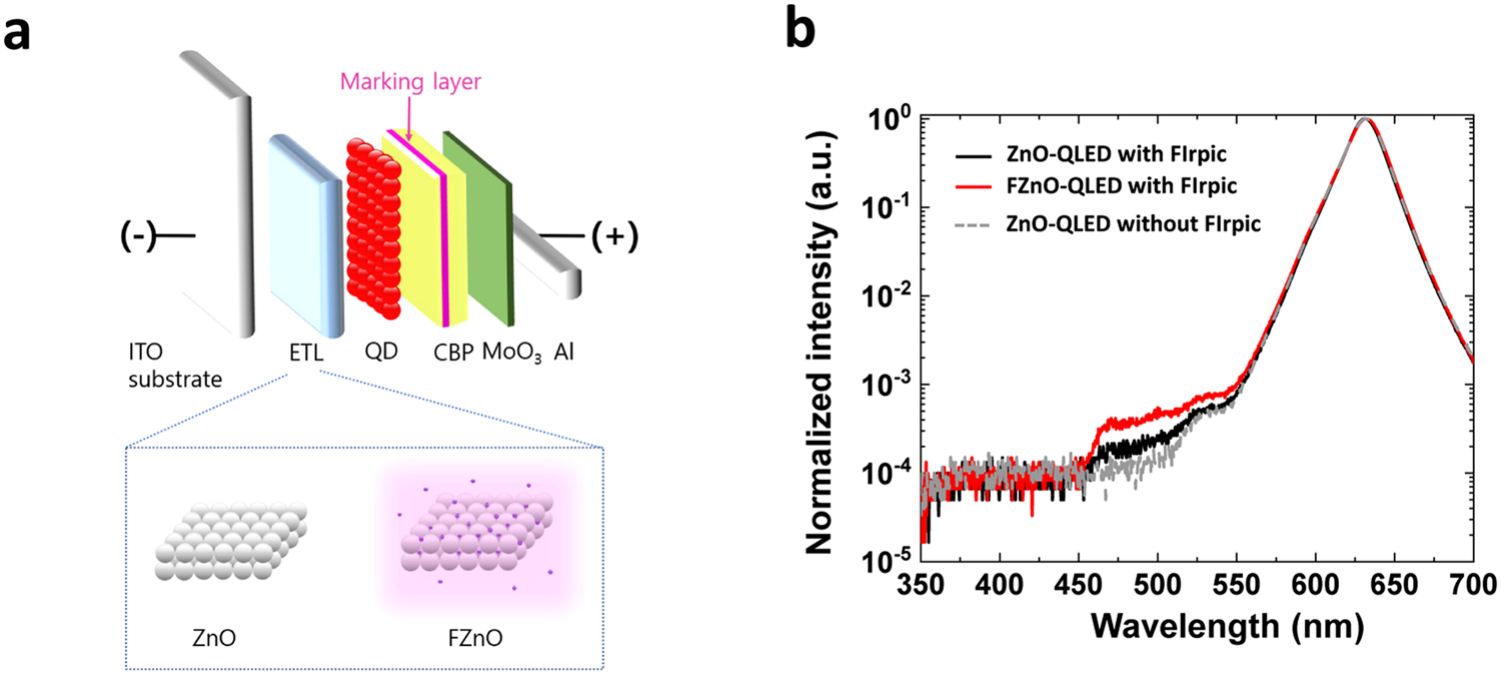
**a** Schematic diagram depicting the structure of the QLEDs with the FIrpic marking layer. **b** EL spectra from the same QLEDs under 20 mA cm^−2^. A spectrum from a ZnO-QLED without the marking layer is also included for comparison

The conclusion that there is a greater electron concentration within the QD EML in case of FZnO may seem at odds with the lower electron currents in the FZnO-EOD *vs.* the ZnO-EOD and is also somewhat counterintuitive from the perspective of charge balance in QLEDs considering that electron transport and injection into the QD is generally easier than that of holes. It should, however, be noted that conductivity in ZnO depends on two factors; the number of intrinsic carriers (electrons) and their mobility, both of which are affected by defect states but in opposite fashions. Therefore, although passivating defect states will reduce the number of electrons in ZnO, it can be expected to improve their mobility due to a reduction in scattering events by defects which may facilitate the arrival of electrons into the QD EML and at the QD/CBP interface. Increasing electron concentration in the QD EML can in turn help facilitate hole injection at the QD/HTL interface by Coulombic interactions [61, 67, 68], and thereby reduce the accumulation of holes at the interface due to the interdependence between the two injection processes. One should note that the lower current density at any given voltage in the *J-V* characteristics of the FZnO-device versus the ZnO-devices in Fig. 2b does not rule out the possibility that electron concentration in the QD EML of the earlier device may be higher, since the *J-V* characteristics could be more strongly governed by carrier concentrations in the ETLs (and where the electron concentration is significantly lower in case of FZnO than in case of ZnO) than by their concentrations in the EML especially also considering that the ETLs are somewhat thicker (40 *vs.* 30 nm). Therefore, to investigate if the FZnO ETL can indeed lead to a higher electron concentration in the QD EML or at the QD/HTL interface, we compare the ETLs in QLEDs that contain a luminescent marking layer at the QD/HTL interface. For this purpose, QLEDs containing a 5 nm thick luminescent marking layer made of CBP doped with 5% bis(2-(4,6-difluorophenyl)pyridinato-C2,N) (picolinato) iridium (III) (FIrpic) are fabricated and tested. The structure of these devices is shown schematically in Fig. 5a and consists of: ITO/ETL/QD/CBP (5 nm)/CBP:FIrpic (5 nm)/CBP (40 nm)/MoO_3_/Al. FIrpic was selected due to its comparable energy structure to CBP which avoids major perturbation of charge distribution at QD/HTL but higher PLQY and thus a more readily detectable luminescence from any exciton recombination within the HTL [20, 21]. Effectively, the marking layer probes changes in the e–h recombination zone due to changes in charge distribution in the device stacks due to the use of FZnO versus ZnO ETLs. Figure 5b shows the EL spectra obtained from these QLEDs as well as from ZnO-QLED without the FIrpic layer to serve as a reference. As can be seen, electroluminescence from FIrpic is observed in the two devices and is higher in case of the FZnO-QLED relative to the ZnO-QLED. The higher FIrpic EL emission points to increased e–h recombination within the HTL in case of the FZnO-QLED device which indicates that the electron concentration in the HTL must be higher indicating that their concentrations in the QD EML and at the QD/HTL interface must also be higher. One may expect the higher electron concentrations in the HTLs in case of the FZnO-QLED to possibly induce new degradation mechanisms, a phenomenon that may be behind the different values of the accelerator coefficients in the FZnO- versus ZnO-device observed in Fig. 3c.

**Fig. 6:**
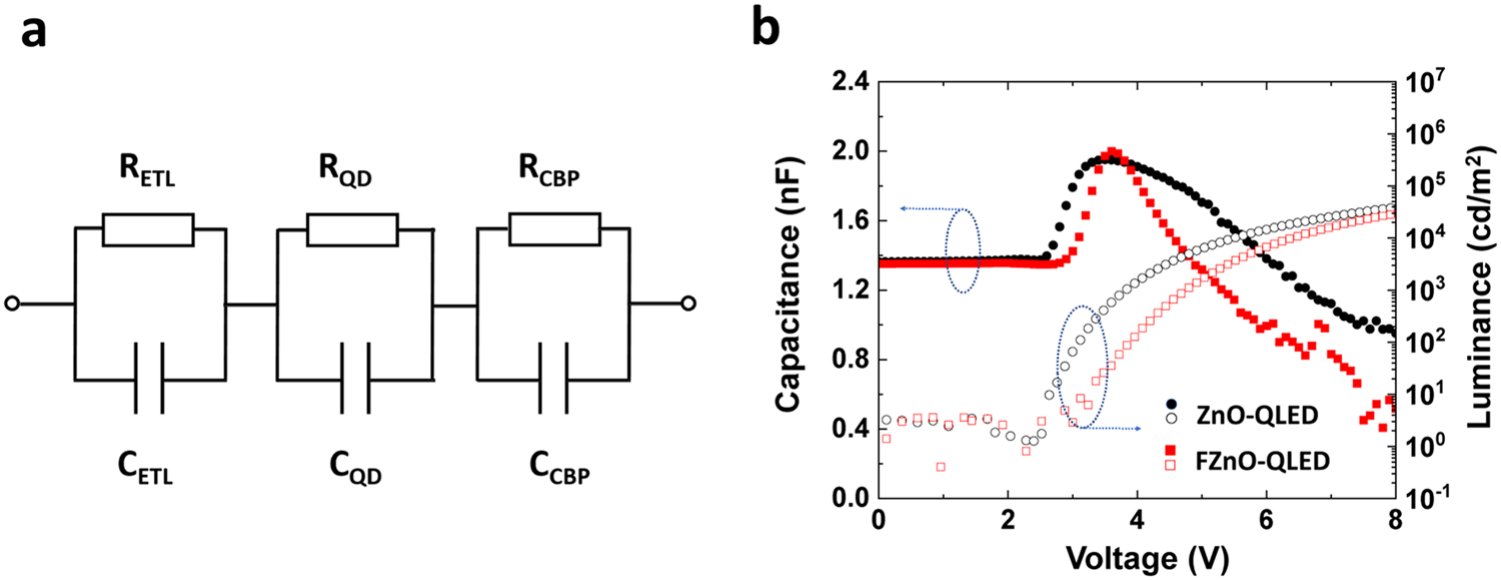
**a** The equivalent circuit model used for the *C-V* analysis. **b** *C-V-L* characteristics of the QLEDs

Capacitance–voltage-luminance (*C-V-L*) measurements can be helpful for probing charge accumulation and annihilation effects within QLEDs [18]. We therefore compare the *C-V-L* characteristics of the FZnO-QLEDs and ZnO-QLEDs to further elucidate the impact of the increased electron concentration in the QD on the interaction with holes in the QD and/or at the QD/CBP interface. The *C-V* characteristics are analyzed with the equivalent circuit model shown in Fig. 6a. The total capacitance across the QLED can be modeled as a set of individual capacitors connected in series, each representing one of the device layers, such that the total reciprocal capacitance of the devices is equal to the sum of each individual layer’s reciprocal capacitance. The geometric capacitance of each layer is calculated by the thickness, cross-sectional area and dielectric constant of each layer using dielectric constant values of 10.5 for ZnO [69], 6.2 for QD [70], and 3 for CBP [71]. Calculations give a total geometric capacitance of 1.23 nF which is comparable to the experimentally measured capacitance of 1.38 nF at voltages below *V*_on_ in both devices as can be shown in Fig. 6b, indicating that charge injection from the contacts at these low voltages is negligible. Above *V*_on_, the capacitance begins to increase pointing to charge injection and accumulation within the device stack, eventually reaching a peak. The slightly lower voltage for the onset of capacitance increase in case of the ZnO-QLED suggests that the presence of a larger number of oxygen vacancies facilitates electron injection from ITO into ZnO. Both QLEDs reach their peak capacitance at around 3.8 V and have comparable peak capacitance values (1.95 and 2.0 nF for ZnO-QLED and FZnO-QLED, respectively) which is close to the geometric capacitance of CBP (1.8 nF) indicating that the electrons reach the QD/HTL interface to form a parallel plate capacitor-like distribution across the CBP layer at this voltage in both devices (The slightly higher peak capacitance of the FZnO-QLED might be due to the higher electron concentration at the QD/HTL interface of this device as was concluded earlier although the difference in capacitance is small and falls within the experimental error and therefore is understandably unreliable to serve as a verification). At biases exceeding 3.8 V the capacitance of both QLEDs decreases, indicating that charges are annihilated by the recombination process therefore leading to the dissipation of the electric double layer [53]. Interestingly, the capacitance decreases much faster with voltage in case of the FZnO-QLED pointing to a faster removal of electrons at the QD/CBP interface. Seen in light of the observations from the marking layer devices above that showed more emission from the FIrpic layer in case of the FZnO-QLED pointing to a higher concentration of electrons at the QD/HTL interface, the *C-V-L* characteristics suggest that enriching the QD/HTL interface with electrons facilitates a faster annihilation of charges at the interface. The ZnO-QLED, in contrast, exhibits a slower charge annihilation process. The mechanism of accumulated charge annihilation for these highly stable QLEDs is schematically depicted in Fig. S6.

In QLEDs, in general, due to the large energy barrier between the valence band maximum of Cd-based QDs and the highest occupied molecular orbital (HOMO) energy level of the HTL, holes accumulate on HTL molecules at the QD/HTL interface. This accumulation of holes can lead to device degradation through a variety of processes [20, 21, 72, 73]. It is therefore quite possible that the increase in the number of electrons at QD/HTL interface as a result of using FZnO ETL results in a decrease in the number of hole that accumulate at the QD/HTL interface, hence the device stability enhancement. This notion is also well supported by the observations from the marking layer devices that showed that using FZnO ETL results in a more efficient e–h recombination at the interface evident in the stronger FIrpic electroluminescence. The results here therefore reveal and underscore the importance of properly managing charge concentrations at the QD/HTL interface for QLED stability.

## Conclusions

Our results demonstrate that chemically treating the ZnO NP ETLs of QLEDs by F plasmas, using carbon tetrafluoride (CF_4_) as a source gas, is found to bring about significant improvements in device EL stability, demonstrating an LT50 at 100 cd m^−2^ of 2,370,000 h in red inverted devices, 47X longer than untreated ZnO. It also leads to 15% higher EQE. XPS, TOF–SIMS and PL measurements reveal that the treatment results in the incorporation of F across the entire bulk of the ZnO film, leading to the passivation of the sub-bandgap states, commonly associated with oxygen vacancies in ZnO films. Electrical measurements on full devices as well as on electron-only devices show a decrease in recombination currents and an earlier onset of trap-filled conduction when the treated films are used, both pointing to the role of F in passivating defect states. TRPL measurements show a longer QD exciton lifetime in devices with FZnO ETLs confirming the defect passivation effect. Additional TRPL measurements under bias reveal that QD exciton lifetime changes differently with current flow in devices with FZnO versus ZnO, suggesting that the F treatment leads to increased electron concentration and trion formation in the QD layers. The higher electron concentration in the QD layer is verified from tests on devices containing a FIrpic luminescent layer, which also show that the use of FZnO increases e–h recombination at the QD/HTL interface. *C-V-L* measurements give further evidence of the increased e–h recombination rate at the QD/HTL interface in QLEDs with FZnO ETL, and of a subsequent decrease in hole accumulation at the interface, the latter known to be a major cause of degradation in these devices, suggesting that the significant stability improvement upon using FZnO is caused by a modulation of electron distribution across the device that leads to a reduction in hole accumulation. The findings provide new insights into the critical roles that optimizing charge distribution across the layers play in influencing stability and present a novel and simple approach for achieving longer lifetimes in QLEDs.

## Supplementary Information

XPS results and surface characterization of films, QD exciton lifetime in EODs, steady state photoluminescence, schematic diagram for exciton dynamics and QLED operation.

Below is the link to the electronic supplementary material.

Supplementary file1 (PDF 1002 KB)
