# Supplementary material for: Significant Lifetime Enhancement in QLEDs by Reducing Interfacial Charge Accumulation via Fluorine Incorporation in the ZnO Electron Transport Layer
Source: Nanomicro Lett. 2022 Nov 4;14:212. doi: 10.1007/s40820-022-00970-x (PMC9636368; doi:10.1007/s40820-022-00970-x)
Supplement: Supplementary file 1 — Supplementary file1 (PDF 1002 KB) [file 40820_2022_970_MOESM1_ESM.pdf]

Supporting Information for

Significant Lifetime Enhancement in QLEDs by Reducing Interfacial Charge Accumulation via Fluorine-Incorporation in the ZnO Electron Transport Layer

Dong Seob Chung[1,*], Tyler Davidson-Hall[1], Giovanni Cotella[2], Quan Lyu[2], Peter Chun[3], and Hany Aziz[1,*]

[1] Department of Electrical and Computer Engineering and Waterloo Institute for Nanotechnology, University of Waterloo, 200 University Avenue West, Waterloo, Ontario N2L 3G1, Canada

[2] Ipswich Research Centre, Huawei Technologies Research & Development (UK) Ltd., Phoenix House (B55), Adastral Park, Ipswich IP5 3RE, UK

[3] Ottawa IC Laboratory, Huawei Canada, 19 Allstate Parkway, Markham, ON L3R 5B4, Canada

*Corresponding authors. E-mail: dschung@uwaterloo.ca (Dong Seob Chung); h2aziz@uwaterloo.ca (Hany Aziz)

S1 XPS Results of Films

Figure S1(a-d) shows XPS spectra of Zn 2p doublet bands in ZnO and FZnO films, C 1s core level electrons in ZnO and FZnO films, O 1s core level electrons in ZnO film and O 1s core level electrons in FZnO film. As can be seen in Fig. S1a, the peaks of the Zn 2p band shift towards higher binding energy in FZnO. This may be ascribed to the high electronegativity of fluorine atoms which pulls the electron cloud from the Zn atoms [S1]. Figure S1b shows the C1s core level electrons spectra from the ZnO and FZnO films. The spectra are generally similar in the two films, and consist of two bands at 285.6 and 289.7 eV, which may be attributed to sp^3 electrons in C-C and C=O bonds [S2]. Figure S1c, d show the O 1s spectra collected from the surface (i.e., before sputter cleaning) of ZnO- and FZnO-films, respectively. The spectra can be deconvoluted into three bands with the same peak positions as in Fig. 1d, e corresponding to O_i, O_{ii} and O_{iii} [S3]. As seen, the highest energy band is proportionally much larger in FZnO relative to ZnO. This may be because the fluorine anions easily interact with hydrogen atoms to form hydroxyl. The fact that this band diminishes after surface cleaning (case of Fig. 1d) suggests that a large fraction of the hydroxyl groups exists on the surface, possibly resulting from increased interactions with atmospheric hydrogen and water molecules due to the presence of F, consistent with observations by Choi et al [S3]. The relative intensity of the middle band from oxygen vacancies is again higher in case of ZnO film relative to its FZnO counterpart, pointing to a larger number of oxygen vacancies.

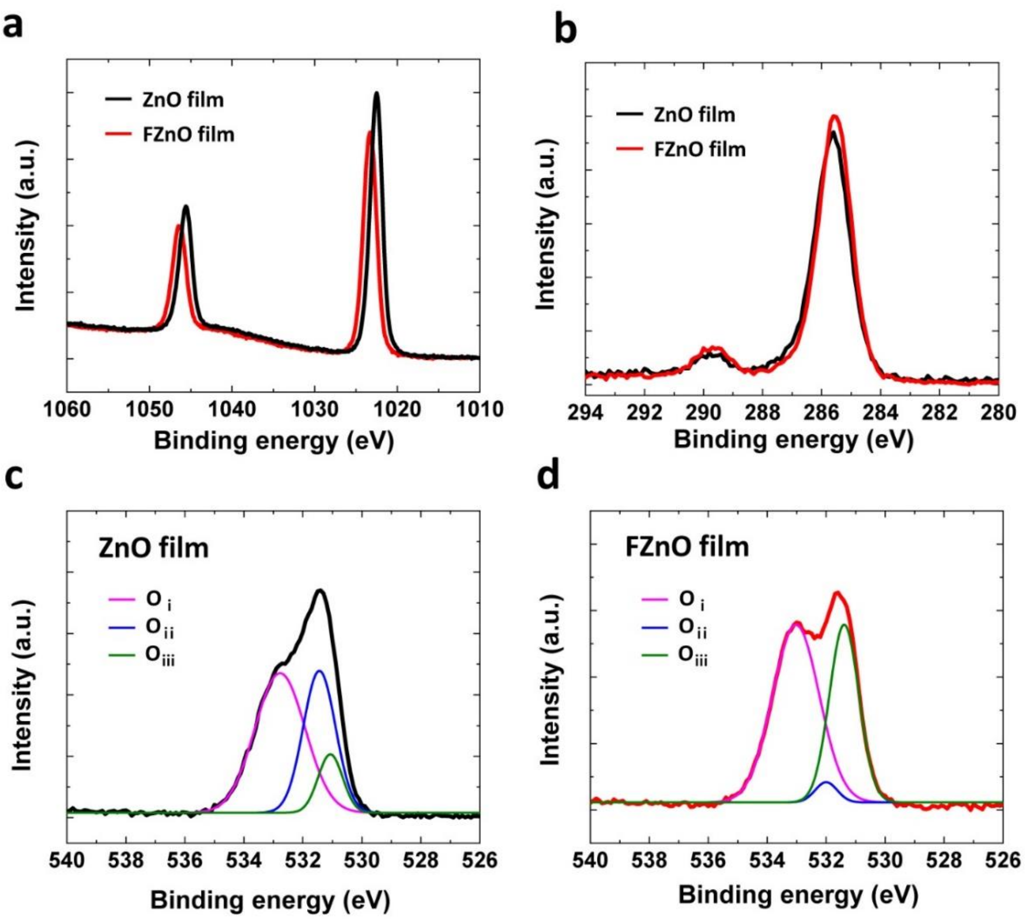

Fig. S1 XPS spectra of **a** Zn 2p doublet bands in ZnO and FZnO films, **b** C 1s core level electrons on a surface of ZnO and FZnO films, and O 1s core level electrons on a surface of **c** ZnO film and **d** FZnO film.

S2 Surface Characterization of Films

To investigate the effect of the CF_4 plasma treatment on the ZnO surface characteristics, contact angle and surface topography measurements are carried out. Figure S2a shows changes in water contact angle and root-mean-square, (RMS) surface roughness of ZnO films as well as in device efficiency and stability after different plasma treatment times. All films are hydrophilic (exhibiting a water contact angle below 90 degree), as seen in Fig. S1a. The CF_4 plasma treatment decreases water contact angle on the surface suggesting the surface becomes more hydrophilic. This may be associated with the presence of a larger number of hydroxyl groups on the surface as suggested by the XPS results in Fig. S1c, d. Changes in RMS surface roughness after various durations of the plasma treatment as measured by Atomic Force Microscopy (AFM) are also shown in Fig. S2a. It can be seen that while 60 s of treatment gives the best device efficiency and stability, the device performance does not vary significantly for treatments between 30 and 120 s, which suggests that the stability enhancement is unlikely to be due to the changes in surface roughness or water contact angle. Figure S2c, d shows the AFM surface topography images of ZnO and FZnO films, respectively. The ZnO film has low RMS roughness of 1.1 nm. As fluorine exposure time increases, the RMS roughness becomes slightly higher but stays below 2.5 nm. The surface topography images show that the FZnO film surface has higher roughness evident in a larger z-range but overall comparable RMS value to the ZnO film. This may be due to c-axis orientation growth of ZnO crystal structure [S4] from fluorine bonding formation.

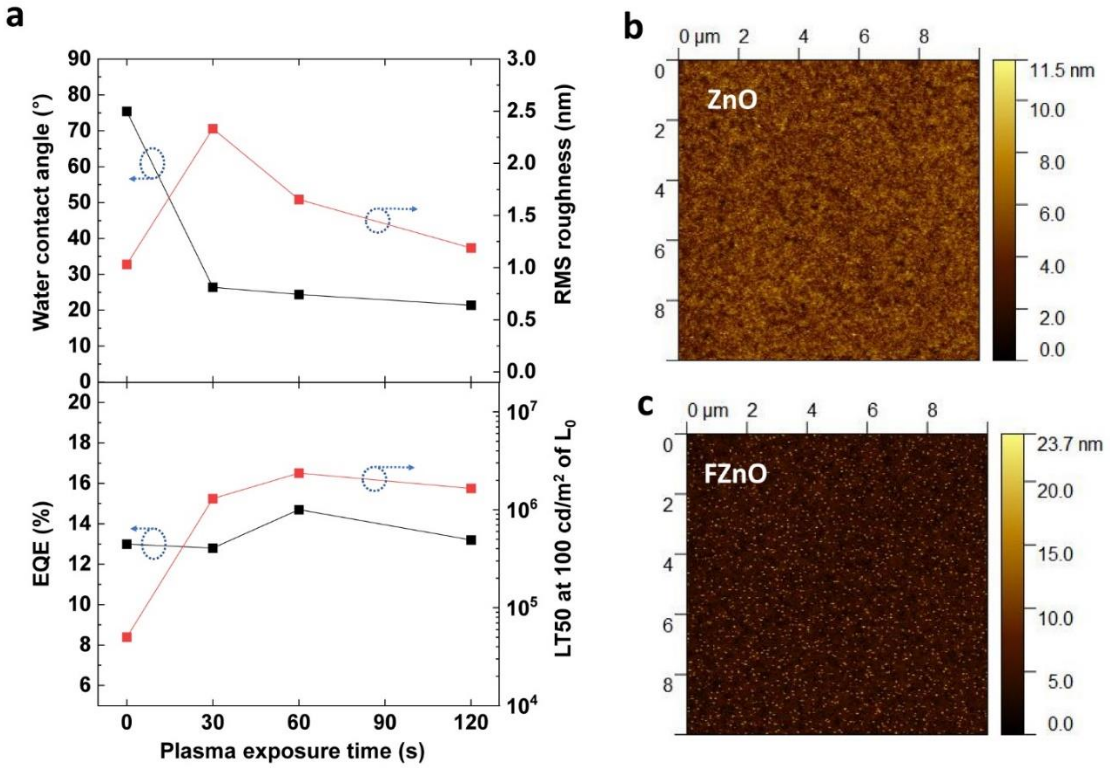

Fig. S2 a Sessile water contact angle on ZnO films surface, root-mean-square (RMS) surface roughness of ZnO films, device efficiency and device stability versus plasma treatment time. **b** and **c** AFM images of ZnO films before and after and 60s of plasma treatment respectively.

S3 QD Exciton Lifetime in EODs, Steady State Photoluminescence Spectral Measurements on Films, and Capacitance-voltage (C-V) Measurements on the EODs

Figure S3 shows TRPL data collected from ZnO-EOD and FZnO-EOD under various forward bias levels (i.e., ITO at a more negative potential relative to Al) and the PL spectra of QD films coated on glass, glass/FZnO and glass/ZnO. The PL decay rate becomes slower (i.e., τ_1 becoming larger, as seen also in Table S2) upon increasing bias in case of ZnO-EOD whereas it changes less and not monotonically (as seen in the initially decreasing and then later increasing τ_1 values in Table S2) in case of FZnO-EOD. The differences point to variations in electron distributions. Figure S3c shows PL spectra collected from QD films coated on FZnO versus ZnO ETLs. Data from QD films coated on glass directly is also provided for comparison. While the PL intensity of the QD layer coated on either ETL is lower than that coated directly on glass as expected, it was higher in case of the FZnO in comparison to ZnO, further verifying the defect passivation effect of the CF_4 plasma treatment. Figure S3d shows *C-V* characteristics of the EODs with ZnO and FZnO ETLs. Both devices show an increase in their capacitance values reaching a value around 1.8 nF which corresponds to the geometric capacitance across the CBP layer as the voltage increases before they start to decrease at higher voltages. This maximum capacitance is reached faster and at lower voltages in case of the FZnO-EOD pointing to higher electron concentration. (Note: The higher 0V capacitance of the EODs *vs.* the full QLEDs can be ascribed to the hold reverse bias conditions applied on the devices prior to the C-V scans which, unlike in the case of the full QLEDs, result in some electron injection from the LiF/Al contact in case of the EODs.)

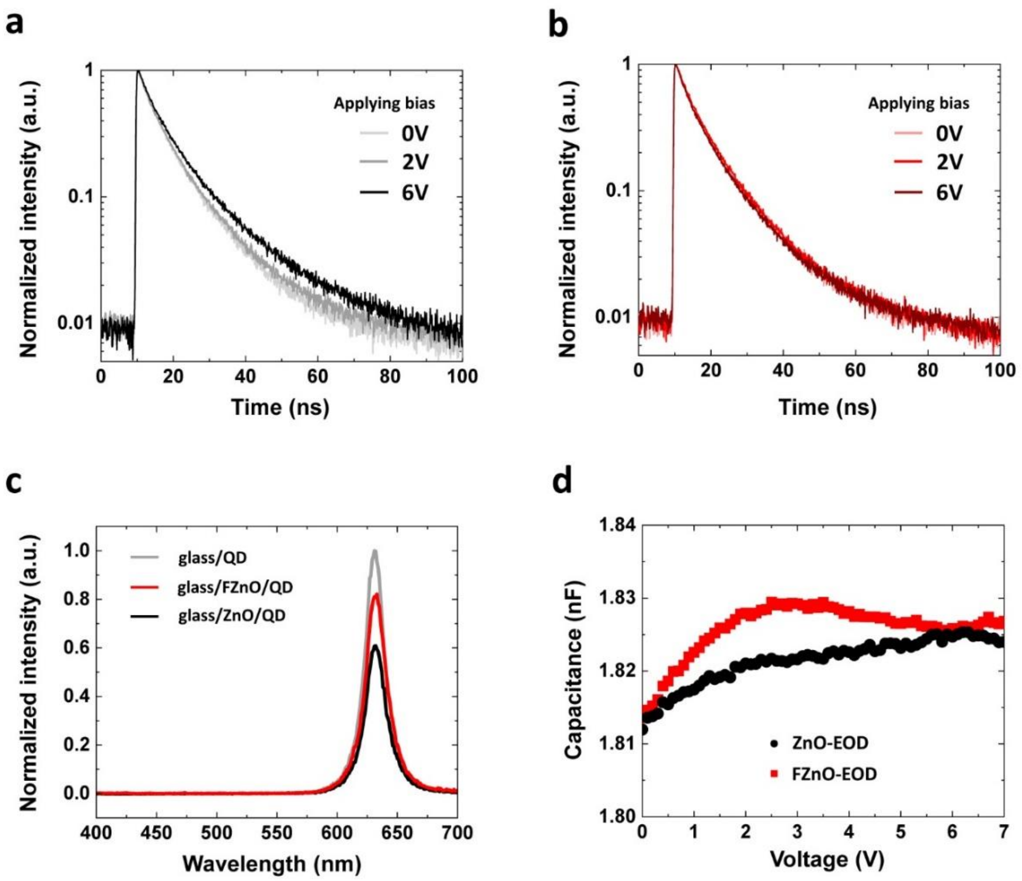

Fig. S3 TRPL results of EODs with **a** ZnO and **b** FZnO with different bias. **c** PL spectra of QD films coated on glass, glass/FZnO, and glass/ZnO. **d** C-V characteristics of the EODs with ZnO and FZnO ETLs.

S4 QD Exciton Lifetime in EODs with Reverse Bias

Figure S4 displays TRPL data collected from ZnO-EOD and FZnO-EOD under various reverse bias levels. Unlike the case of forward bias, the PL decay rate does not change with reverse bias. This suggests that changes in TRPL signal observed under forward bias are induced by changes in carrier concentration (electrons) as opposed to by changes in electric fields.

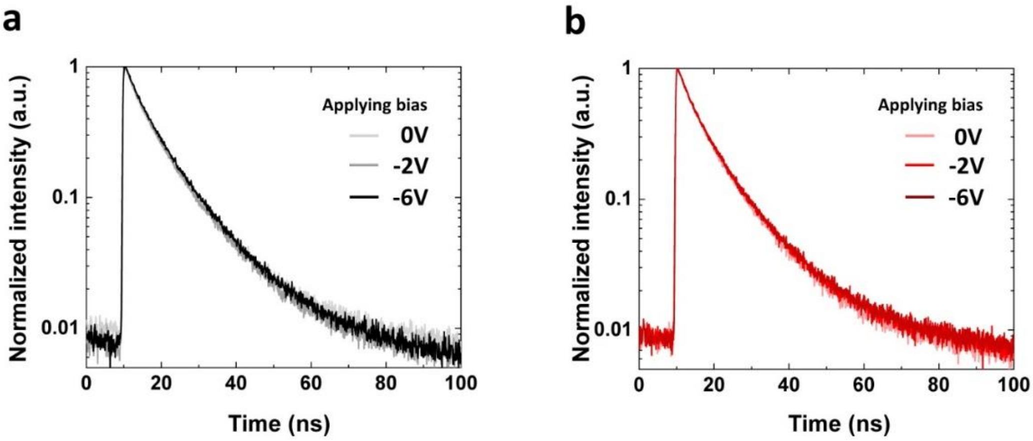

Fig. S4 TRPL results of EODs with **a** ZnO and **b** FZnO with reverse bias.

S5 Schematic Illustration Showing Changes in Charge Distribution and Its Effect on Photo-generated Excitons (Trion Formation) in Case of FZnO- *vs.* ZnO-EOD

Figure S5 schematically depicts changes in charge distribution due to the passivation of oxygen vacancies in case of the FZnO ETL. The reduced oxygen vacancies lead to a higher electron concentration at the QD, leading to more trion formation under UV-excitation. Interactions between injected charges and photo-generated excitons lead to the changes in the QD exciton lifetime with forward bias observed in Fig. 4d.

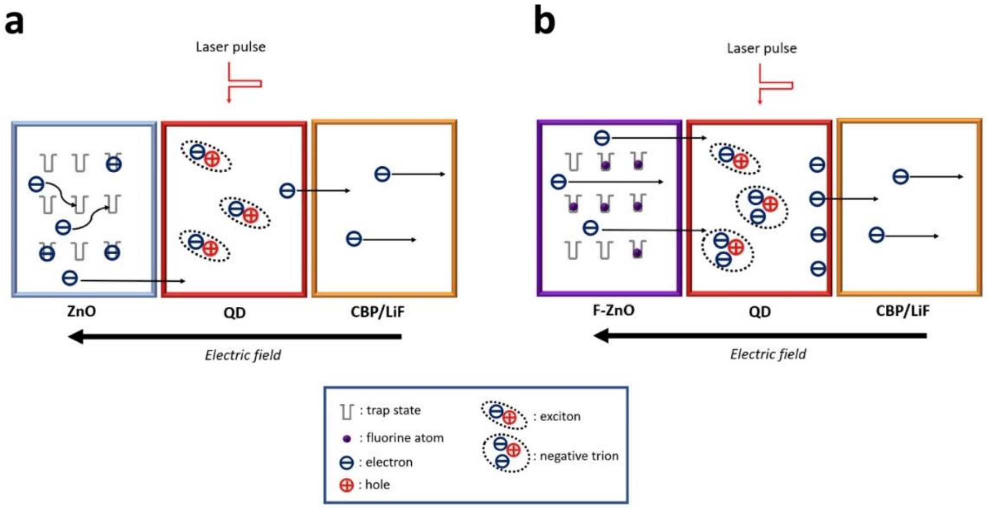

Fig. S5 Schematic images of exciton dynamics in EODs with **a** ZnO and **b** FZnO.

S6 Schematic Illustration Showing Changes in Charge Distribution and Its Effect on Hole Accumulation at the QD/HTL Interface in case of FZnO- *vs.* ZnO-QLED

Figure S6 schematically depicts changes in charge distribution due to the passivation of oxygen vacancies in case of the FZnO ETL. The reduced oxygen vacancies lead to a higher electron concentration at the QD/HTL interface, and thus reduces hole accumulation and as a result improves device stability [S5, S6].

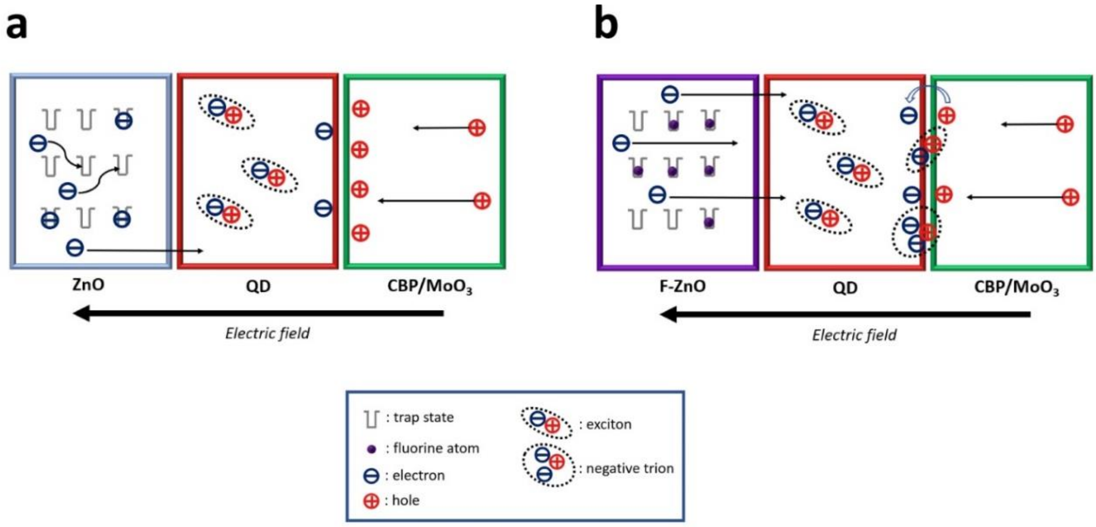

Fig. S6 Schematic images of device operation in QLEDs with **a** ZnO and **b** FZnO.

Table S1 Comparison of the stability performance of the QLEDs reported here with other highly stable devices with CdSe QDs via ETL modifications [S5, S7-S17]. LT50s not reported in the original work are estimated using Eq. (5) and an acceleration factor 1.8

ETLs	QDs	HTLs	Lifetime at L_0 of 100 cd/m² (hours)	Ref
Fluorine:ZnO	CdZnSe/ZnSe/CdZnS/ZnS	CBP	LT50 : 2,370,000	This work
Mg:ZnO	CdSe/ZnS	TFB	LT50 : 510,000	[7]
ZnLiMgO@MgO	CdZnSe/ZnS	TFB	N/A (LT95 : 381,000)	[8]
Spiro-OMeTAD/ZnMgO	CdSe/ZnS	TFB	LT50 : 24,400	[9]
TBS-PBO/ZnMgO	CdSe/CdS/ZnS	Poly-TPD	LT50 : 70,000	[10]
Rb_2Co_3:ZnO	CdZnSeS/ZnS	TCTA	LT50 : 11,000	[11]
PMMA/ZnO	CdSe/CdS	Poly-TPD	LT50 : 420,000	[12]
PEI:ZnO	CdZnSe/ZnSe/CdZnS/ZnS	CBP	LT50 : 154,000	[5]
Y:ZnO	CdSe/CdZnS	CBP	LT50 : 3,700	[13]
Li:ZnO	CdSe/ZnS	TCTA/NPB	N/A (LT95 : 11,000)	[14]
Ethanolamine:ZnO	CdSe/CdS/CdZnS	TFB	LT50 : 2,000,000	[15]
SnO_2	CdSe/ZnS	TFB	N/A (LT95 : 20,200)	[16]
TiO_2	CdSe/CdS/ZnS	CBP/TCTA	N/A (LT95 : 0.3)	[17]

The abbreviations in Table S1

CBP: 4,4′-Bis(N-carbazolyl)-1,1′-biphenyl

TFB: Poly(9,9-dioctylfluorene-alt-N-(4-sec-butylphenyl)-diphenylamine)

ZnLiMgO@MgO: ZnLiMgO nanoparticles with magnesium oxide shells

Spiro-OMeTAD: 2,2',7,7'-Tetrakis[N,N-di(4-methoxyphenyl)amino]10-9,9'-spirobifluorene

TBS-PBO: tert-butyldimethylsilyl-poly(p-phenylene benzobisoxazole)

Poly-TPD: Poly[N,N′-bis(4-butylphenyl) -N,N′-bis(phenyl)-benzidine]

TCTA: tris(4-carbazoyl-9-ylphenyl)amine

PMMA: Poly(methyl methacrylate)

PEI: Polyethylenimine

NPB: N,N′-Di(1-naphthyl)-N,N′-diphenyl-(1,1′-biphenyl)-4,4′-diamine

Table S2 τ and A components extracted from the tri-exponential decay curves. The first two rows correspond to the measurement under reverse bias

	Bias	τ_1	A_1	τ_2	A_2	τ_3	A_3
**ZnO-EOD**	-6V	2.65	0.30	7.63	0.64	54.88	0.05
	-2V	2.63	0.32	9.22	0.65	52.66	0.05
	0V	2.77	0.31	8.78	0.66	55.94	0.04
	2V	2.81	0.31	7.99	0.66	56.32	0.03
	3V	2.90	0.33	8.95	0.65	58.77	0.03
	4V	3.04	0.35	9.02	0.62	53.35	0.04
	5V	3.06	0.35	9.11	0.62	53.53	0.05
	6V	3.17	0.34	9.55	0.62	52.75	0.05
**FZnO-EOD**	-6V	3.26	0.33	8.63	0.60	17.93	0.06
	-2V	3.21	0.37	9.22	0.62	27.21	0.02
	0V	3.19	0.43	9.33	0.57	79.62	0.02
	2V	3.02	0.39	8.51	0.60	69.29	0.02
	3V	2.81	0.33	8.56	0.65	63.56	0.03
	4V	2.87	0.35	8.86	0.62	55.88	0.04
	5V	2.90	0.35	9.05	0.62	59.5	0.05
	6V	3.08	0.34	9.45	0.62	64.05	0.05

Supplementary References

[S1] Y. Xu, B. Bo, X. Gao, Z. Qiao, Passivation effect on ZnO films by SF_6 plasma treatment. Crystals **9**(5), 236 (2019). https://doi.org/10.3390/cryst9050236

[S2] A. Dolgov, D. Lopaev, C.J. Lee, E. Zoethout, V. Medvedev et al., Characterization of carbon contamination under ion and hot atom bombardment in a tin-plasma extreme ultraviolet light source. Appl. Surface Sci. **353**, 708-713 (2015). https://doi.org/10.1016/j.apsusc.2015.06.079

[S3] Y.-J. Choi, K.-M. Kang, H.-H. Park, Anion-controlled passivation effect of the atomic layer deposited ZnO films by f substitution to O-related defects on the electronic band structure for transparent contact layer of solar cell applications. Solar Energy Mater. Solar Cells **132**, 403-409 (2015). https://doi.org/10.1016/j.solmat.2014.09.029

[S4] S.-H. K. Park, Y.E. Lee, Controlling preferred orientation of ZnO thin films by atomic layer deposition. J. Mater. Sci. **39**(6), 2195-2197 (2004). https://doi.org/10.1023/B:JMSC.0000017786.81842.ae

[S5] D.S. Chung, T. Davidson-Hall, H. Yu, F. Samaeifar, P. Chun et al., Significant enhancement in quantum-dot light emitting device stability via a ZnO: Polyethylenimine mixture in the electron transport layer. Nanoscale Adv. **3**(20), 5900-5907 (2021). https://doi.org/10.1039/D1NA00561H

[S6] T. Davidson-Hall, H. Aziz, Significant enhancement in quantum dot light-emitting device stability via a cascading hole transport layer. ACS Appl. Mater. Interfaces **12**(14), 16782-16791 (2020). https://doi.org/10.1021/acsami.9b23567

[S7] Z. Chen, Q. Su, Z. Qin, S. Chen, Effect and mechanism of encapsulation on aging

characteristics of quantum-dot light-emitting diodes. Nano Res. **14**(1), 320-327 (2021). https://doi.org/10.1007/s12274-020-3091-3

[S8] D. Liu, S. Cao, S. Wang, H. Wang, W. Dai et al., Highly stable red quantum dot light-emitting diodes with long T_{95} operation lifetimes. J. Phys. Chem. Lett. **11**(8), 3111-3115 (2020). https://doi.org/10.1021/acs.jpclett.0c00836

[S9] W. Zhang, Q. Zhang, Y. Zhang, F. Li, C. Chang et al., CdSe/ZnS quantum-dot light-emitting diodes with Spiro-OMeTAD as buffer layer. IEEE Trans. Electr. Devices **66**(11), 4901-4906 (2019). https://doi.org/10.1109/TED.2019.2937788

[S10] X. Jin, C. Chang, W. Zhao, S. Huang, X. Gu, Q. Zhang, F. Li, Y. Zhang, Q. Li. et al., Balancing the electron and hole transfer for efficient quantum dot light-emitting diodes by employing a versatile organic electron-blocking layer. ACS Appl. Mater. Interfaces **10**(18), 15803-15811 (2018). https://doi.org/10.1021/acsami.8b00729

[S11] Y. Lee, H.-M. Kim, J. Kim, J. Jang, Remarkable lifetime improvement of quantum-dot light emitting diodes by incorporating rubidium carbonate in metal-oxide electron transport layers. J. Mater. Chem. C **7**(32), 10082-10091 (2019). https://doi.org/10.1039/C9TC02683E

[S12] X. Dai, Z. Zhang, Y. Jin, Y. Niu, H. Cao et al., Solution-processed, high-performance light-emitting diodes based on quantum dots. Nature **515**(7525), 96-99 (2014). https://doi.org/10.1038/nature13829

[S13] Y. Lee, B.G. Jeong, H. Roh, J. Roh, J. Han et al., Enhanced lifetime and efficiency of red quantum dot light-emitting diodes with Y-doped ZnO sol–gel electron-transport layers by reducing excess electron injection. Adv. Quantum Techn. **1**(1), 1700006 (2018). https://doi.org/10.1002/qute.201700006

[S14] J. Jing, L. Lin, K. Yang, H. Hu, T. Guo et al., Highly efficient inverted quantum dot light-emitting diodes employing sol-gel derived Li-doped ZnO as electron transport layer. Organic Electron. **103**, 106466 (2022). https://doi.org/10.1016/j.orgel.2022.106466

[S15] K. Noh, M. Kim, S.-H. Lee, H.-S. Yun, T.-H. Lim et al., Effect of ethanolamine passivation of ZnO nanoparticles in quantum dot light emitting diode structure. Curr. Appl. Phys. **19**(9), 998-1005 (2019). https://doi.org/10.1016/j.cap.2019.05.016

[S16] M. Chen, X. Chen, W. Ma, X. Sun, L. Wu et al., Highly stable SnO_2-based quantum-dot light-emitting diodes with the conventional device structure. ACS Nano (2022). https://doi.org/10.1021/acsnano.2c02912

[S17] W. Ji, P. Jing, J. Zhao, X. Liu, A. Wang et al., Inverted CdSe/CdS/ZnS quantum dot light emitting devices with titanium dioxide as an electron-injection contact. Nanoscale **5**(8), 3474-3480 (2013). https://doi.org/10.1039/C3NR34168B
